# p21-activated kinase is involved in the sporulation, pathogenicity, and stress response of *Arthrobotrys oligospora* under the indirect regulation of Rho GTPase-activating protein

**DOI:** 10.3389/fmicb.2023.1235283

**Published:** 2023-09-14

**Authors:** Meichen Zhu, Yankun Liu, Xuewei Yang, Lirong Zhu, Yanmei Shen, Shipeng Duan, Jinkui Yang

**Affiliations:** ^1^State Key Laboratory for Conservation and Utilization of Bio-Resources and Key Laboratory for Microbial Resources of the Ministry of Education, Yunnan University, Kunming, China; ^2^School of Life Sciences, Yunnan University, Kunming, China

**Keywords:** p21-activated kinase, Rho GTPase-activating protein, conidiation, trap formation, *Arthrobotrys oligospora*

## Abstract

The p21-GTPase-activated protein kinases (PAKs) participate in signal transduction downstream of Rho GTPases, which are regulated by Rho GTPase-activating proteins (Rho-GAP). Herein, we characterized two orthologous Rho-GAPs (AoRga1 and AoRga2) and two PAKs (AoPak1 and AoPak2) through bioinformatics analysis and reverse genetics in *Arthrobotrys oligospora*, a typical nematode-trapping (NT) fungus. The transcription analyses performed at different development stages suggested that *Aopaks* and *Aorga1* play a crucial role during sporulation and trap formation, respectively. In addition, we successfully deleted *Aopak1* and *Aorga1* via the homologous recombination method. The disruption of *Aopak1* and *Aorga1* caused a remarkable reduction in spore yield and the number of nuclei per cell, but did not affect mycelial growth. In ∆*Aopak1* mutants, the trap number was decreased at 48 h after the introduction of nematodes, but nematode predatory efficiency was not affected because the extracellular proteolytic activity was increased. On the contrary, the number of traps in ∆*Aorga1* mutants was significantly increased at 36 h and 48 h. In addition, *Aopak1* and *Aorga1* had different effects on the sensitivity to cell-wall-disturbing reagent and oxidant. A yeast two-hybrid assay revealed that AoPak1 and AoRga1 both interacted with AoRac, and AoPak1 also interacted with AoCdc42. Furthermore, the *Aopaks* were up-regulated in ∆*Aorga1* mutants, and *Aorga1* was down-regulated in ∆*Aopak1* mutants. These results reveal that AoRga1 indirectly regulated AoPAKs by regulating small GTPases.

## 1. Introduction

The p21-GTPase-activated protein kinase (PAK) family is present in all eukaryotes (Keniry and Sprague, 2003). The PAK proteins have a conserved N-terminal domain (Cdc42/Rac interactive binding, CRIB) required for binding to the Rho GTPases Cdc42 and Rac, and are regulated by an autoinhibitory mechanism involving the kinase and CRIB domains (Boyce and Andrianopoulos, 2011). This family is identified by sequence similarity with the kinase and CRIB domains, and divided into two groups (Cotteret et al., 2003; Keniry and Sprague, 2003; Molli et al., 2009). *Saccharomyces cerevisiae* contains three members of the PAK family, Ste20, Cla4, and Skm1 (Martin et al., 1997), each of which share some conserved functions and also perform distinct roles. The PAK family regulates cell and actin polarization throughout the cell cycle of *S. cerevisiae* (Holly and Blumer, 1999). Furthermore, the PAKs are required for the proper establishment of cell polarity in *Schizosaccharomyces pombe* (Qyang et al., 2002) and *Cryptococcus neoformans* (Nichols et al., 2004). In *Talaromyces marneffei*, PakA (Homologous protein of AoPak2) contributes to polarity establishment during conidial germination and polarization growth (Boyce and Andrianopoulos, 2007). In *C. neoformans*, Pak1 is involved in mating and virulence (Nichols et al., 2004). In addition, Ste20 protein kinases can trigger hyphal formation in the pathogenic fungus *Candida albicans* (Leberer et al., 1996). In *Ustilago maydis*, the deletion of *cla4* causes defects in pathogenicity and the filamentous growth of the mating reaction (Leveleki et al., 2004). In *Magnaporthe grisea*, *chm1* (*Cla4* homolog in *M. grisea*) and *mst20* (*M. grisea Ste20* homolog) encode two PAK proteins with distinct functions, *mst20* is dispensable for plant infection, and *chm1* plays a critical role in appressorium formation and penetration in *M. grisea* (Li et al., 2004). Therefore, the PAK family play important roles in fungal conidial germination, pathogenicity, and spore production, especially in the establishment of polarity, and their specific functions vary by species.

The PAK family act as downstream effectors of the Rho GTPases in a variety of morphogenic processes. Rho GTPase family proteins include Rho1, Rho2, Rho3, Rho4, Rho5, Cdc42, and Rac (Mosaddeghzadeh and Ahmadian, 2021). Rho GTPases act as a molecular switch, toggling between an active GTP-bound form and an inactive GDP-bound form, and are involved in diverse cellular functions via the negative regulation of Rho GTPase activating proteins (Rho-GAPs; Bassilana et al., 2005; Ye et al., 2014). Three Rho-GAPs have been functionally studied in *S. cerevisiae* (Roumanie et al., 2001). Rho-GAPs are required for a variety of processes related to signal transduction in cell development. In *M. oryzae*, *Morga2* to *Morga7* (coding Rho-GAPs) are dispensable for conidiation, vegetative growth, appressorial formation and pathogenicity, but *Molrg1* and *Morga1* (homologous genes of *rho-GAPs*) are crucial for pathogenicity (Ye et al., 2014). In *C. albicans*, the deletion of the *Cargd1* increases filamentous growth, and cells lacking *Cargd1* present longer germ tubes, whereas the overexpression of *rgd1* restricts hyphae growth (Ness et al., 2010). In addition, Cdc42 GAP (Cdc42 GTPase-activating protein, also known as p50RhoGAP or ARHGAP1) plays an important role in regulating mammalian cell genomic stability (Wang et al., 2007), and p200RhoGAP, a member of the Rho-GAP family, can mediate cross-talks between Ras- and Rho-regulated signaling pathways in cell growth regulation (Shang et al., 2007). Therefore, Rho-GAPs are required for mycelial development in various fungi, but the function and the mechanism underlying Rho-GAPs in Rho-mediated signaling pathways are still unknown in nematode-trapping (NT) fungi.

Plant-parasitic nematodes are a source of serious potential damage in agriculture and horticulture (Phani et al., 2021; Zhu et al., 2022a). Biological control stands out among the many nematode control methods, including chemical control and traditional practices of control (solarization, planting trap crops before sowing, in rotation, or after cultivation), with advantages of high efficiency, low toxicity and eco-friendliness (Philbrick et al., 2020; Sushil et al., 2022; Tapia-Vazquez et al., 2022). NT fungi are a promising source of biocontrol that can be employed to develop specialized structures called “traps” to capture, kill, and consume nematodes (Ji et al., 2020). *Arthrobotrys oligospora*, a representative NT fungus, has been sequenced for its whole genome and proteome (Yang et al., 2011). Recent studies have shown that signal transduction pathways play a vital role in hyphal growth and trap formation in *A. oligospora*. Rho-GAPs, small GTPases, and PAKs are involved in the transmission of signals between extracellular and intracellular regions, which is involved in the regulation of the cAMP-PKA pathway (Swaminathan et al., 2014; Yang et al., 2022) and mitogen-activated protein kinase (MAPK) cascades (Martin et al., 1997; Cansado et al., 2010). Recently, we have demonstrated that AoRac and AoCdc42 play a crucial role in hypha growth, lipid accumulation, DNA damage, sporulation, trap formation, pathogenicity, and stress response (Yang et al., 2022). Subsequently, the cAMP-PKA signaling pathway has been proven to be involved in hyphal growth, sporulation, trap morphogenesis, stress tolerance, and autophagy in *A. oligospora* (Chen et al., 2022; Zhu et al., 2022b). In addition, several genes are related to MAPK cascades, such as *mkk1* (coding a MAP kinase kinase) and *bck1* (coding a MAP kinase kinase kinase; Xie et al., 2021), *ste7* (coding a MAP kinase kinase) *and fus3* (coding a MAP kinase; Chen et al., 2021; Xie et al., 2023), *hog1* (coding a MAP kinase; Kuo et al., 2020), and *ime2* (coding a MAP kinase; Xie et al., 2020), which all participate in the development and pathogenicity of *A. oligospora*.

In this study, we identified two Rho-GAPs and two PAKs in *A. oligospora* using orthologous proteins in *S. cerevisiae* as reference sequences, and we have elucidated function and possible mechanisms of *Aorga1* and *Aopak1* in *A. oligospora* via phenotypic analysis, interaction protein verification, and expression pattern analysis. Our results indicate that PAKs are involved in the sporulation, pathogenicity, and stress resistance of *A. oligospora* under the indirect regulation of Rho-GAPs.

## 2. Materials and methods

### 2.1. Fungal strains and culture conditions

The wild-type (WT) strain *A. oligospora* (ATCC 24927) was maintained on potato dextrose agar (PDA) medium at 28°C, and the derived mutants were incubated on a PDA medium supplement with 200 μg/mL hygromycin. TGA (1% tryptone, 1% glucose, 2% agar) and TYGA (TGA with 0.5% yeast extracts and 1% molasses) media were used for analyzing the phenotypic traits. Sporulation and trap induction were performed on corn meal yeast extract (CMY) and water agar (WA) medium, respectively. The nematode *Caenorhabditis elegans* (strain N2) was maintained on oatmeal medium at room temperature for the bioassay. The mutant strains used in this study are listed in Supplementary Table S1.

### 2.2. Bioinformatic analysis

The protein sequences of AoPak1 (AOL_s00004g340), AoPak2 (AOL_s00079g352), AoRga1 (AOL_s00110g81), and AoRga2 (AOL s00076g167) were identified in *A. oligospora* using the orthologs from the model fungus *S. cerevisiae*. The candidate sequences were chosen based on an E-value of ≤1e^−10^. Similarly, we retrieved homologous proteins from *Magnaporthe oryzae*, *Neurospora crassa*, *Aspergillus nidulans*, *Beauveria bassiana* and five NT fungi, including *Dactylella cylindrospora*, *Drechslerella stenobrocha*, *Drechslerella brochopaga*, *Arthrobotrys flagrans*, and *Arthrobotrys entomopaga*. The sequence similarities between orthologous PAKs and Rho-GAPs from different fungi were aligned with Geneious 4.8.5 software. The phylogenetic trees were constructed using MEGA 7.0 with the neighbor-joining method, the JTT + I + G substitution model with 1,000 bootstrap replicates. The phylogenetic trees were visualized using FigTree v1.4.2. The conserved domains were predicted using Pfam 35.0 (Zhu et al., 2022b).^1^

### 2.3. Gene disruption and southern blotting analysis

The targeted genes were disrupted using the homologous recombination method as described previously (Colot et al., 2006). Upstream and downstream 2000 bp fragments of the target genes were amplified from *A. oligospora* DNA. The selection marker gene *hph* was amplified from the pCSN44 plasmid. Three fragments were inserted into the pRS426 plasmid to form a fusion vector. The recombinant fragments were amplified by PCR and transformed into *A. oligospora* protoplasts in a PEG-mediated manner, as described previously (Tunlid et al., 1999; Zhu et al., 2023). Transformants were cultured on PDAS medium with 200 μg/mL hygromycin B and verified by PCR, and positive transformants were further confirmed by Southern blot analysis according to the instructions of the North2South Chemiluminescent Hybridization Detection Kit (Pierce, Rockford, United States). The primers are listed in Supplementary Table S4.

### 2.4. Comparison of mycelial growth and conidiation

The WT and mutant strains were activated on PDA medium for 5 days; then, colonies with a diameter of 9 mm were, respectively, inoculated on TG, PDA, and TYGA media for 7 days at 28°C. The colony diameter was recorded every 24 h, and photographs were taken on the seventh day to record morphology. For sporulation analysis, the activated WT and mutant strains were inoculated on CMY medium for 15 days at 28°C, sterile water (20 mL) was added to the cultures before they were shaken with glass beads to separate spores from mycelia, and then the cultures were filtered to obtain a conidial suspension. Then, the numbers of conidia per microliter of suspension were measured using a counter. The number of spores per cm^2^ medium can be used as an estimate of the conidial yield (Zhu et al., 2022b).

### 2.5. Trap induction and pathogenicity analysis

The suspensions containing 20,000 conidia of WT and mutants were inoculated separately on WA medium and incubated at 28°C for 3–4 days until the mycelium covered the entire plate. In total, 200\u00B0*C. elegans* were added to each plate to induce trap formation. The same number of nematodes as in the experimental group was added to the WA plate that was not inoculated with the strain to indicate the number of nematodes that died naturally, which was indicated by “uninfected.” We observed the trap morphology, and quantified the numbers of traps and nematode mortality at 12 h intervals.

### 2.6. Analysis of extracellular proteolytic activity

The 9 mm colony discs of WT and mutant strains were added to PD broth medium with skimmed milk (8%) and incubated at 180 rpm and 28°C for 5 days. The fermentation solution was filtered under sterile conditions to obtain the liquid supernatant. The qualitative assessment of protease activity was carried out by adding fermentation broth to the casein skimmed milk plates, and the quantitative analysis of protease activity was carried out as previously described (Yang et al., 2021; Li et al., 2023). All experiments were performed with at least three repetitions.

### 2.7. Quantification of the nucleus number

For the staining of nuclei, 10 μg/mL DAPI (Sigma-Aldrich) was added to the 5-day-old mycelia of WT and mutants for 10 min, followed by the addition of 20 μL calcofluor white (CFW,10 μg/mL, Sigma-Aldrich). These were then photographed with a fluorescent microscope after 10 min of staining, followed by counting the number of nuclei in each cell using ImageJ.

### 2.8. Response to chemical stressors

The 9 mm fungal discs of activated WT and mutant strains were inoculated on TG plates supplemented with different concentrations of stress reagents, and the colony diameters were measured after 7 days of incubation at 28°C. The diameter of the colony cultured on TG medium without stress reagents was used as a control to calculate the relative growth inhibition (RGI; Zhen et al., 2018).

### 2.9. Reverse transcription–polymerase chain reaction (RT-PCR) analysis

The mycelial samples used for the transcription analyses of genes related to proteases, sporulation, cell wall synthesis, and oxidative stress response were collected from PDA combined with nematodes, CMY, TG with Congo red (0.06 mg/mL) or menadione (0.07 mM), respectively. Total RNA was isolated using an RNA extraction kit (Axygen, Jiangsu, Suzhou) and reverse-transcribed cDNA using a PrimeScript^RT^ reagent kit (TaKaRa, Japan) according to the manufacturer’s instructions. The transcriptional levels of genes were detected using the LightCycler 480 SYBR green I master mix (Roche, Basel, Switzerland), and β-tubulin (*AOL_s00076g640*) was used as the internal control. The relative transcript level of each gene was calculated using the 2^−∆∆CT^ method (Livak and Schmittgen, 2001). All primers used for RT-PCR are listed in Supplementary Table S4.

### 2.10. Yeast two-hybrid (Y2H) assay

The coding sequences of AoRga1 and AoPak1 were cloned into the pGBKT7 vector as bait, respectively. The encoding sequences of small GTPases AoCdc42, AoRas, and AoRac were inserted separately into the pGADT7 vector as prey. The cDNA is used as the template for PCR amplification and the primers are listed in Supplementary Table S4. The pGBKT7 and pGADT7 fusion plasmids were co-transformed into the Y2H Gold (Weidi, Shanghai, China) competent cells and inoculated on synthetic dropout medium (SD/−Trp/−Leu and SD/−Trp/−Leu/–His/−Ade). The substrate X-*α*-gal was added to the SD/−Ade/–His/−Leu/−Trp medium to detect the *α*-galactosidase activity.

### 2.11. Statistical analysis

All experimental data have been presented as the mean ± standard deviation (SD) of three biological repetitions. The multiple T-test was performed using Prism 8.0 (GraphPad, San Diego, CA, United States) to statistically evaluate the differences between treatments. *p* < 0.05 was considered significant.

## 3. Results

### 3.1. Pak and Rho-GAP are conserved in filamentous fungi

**Figure 1:**
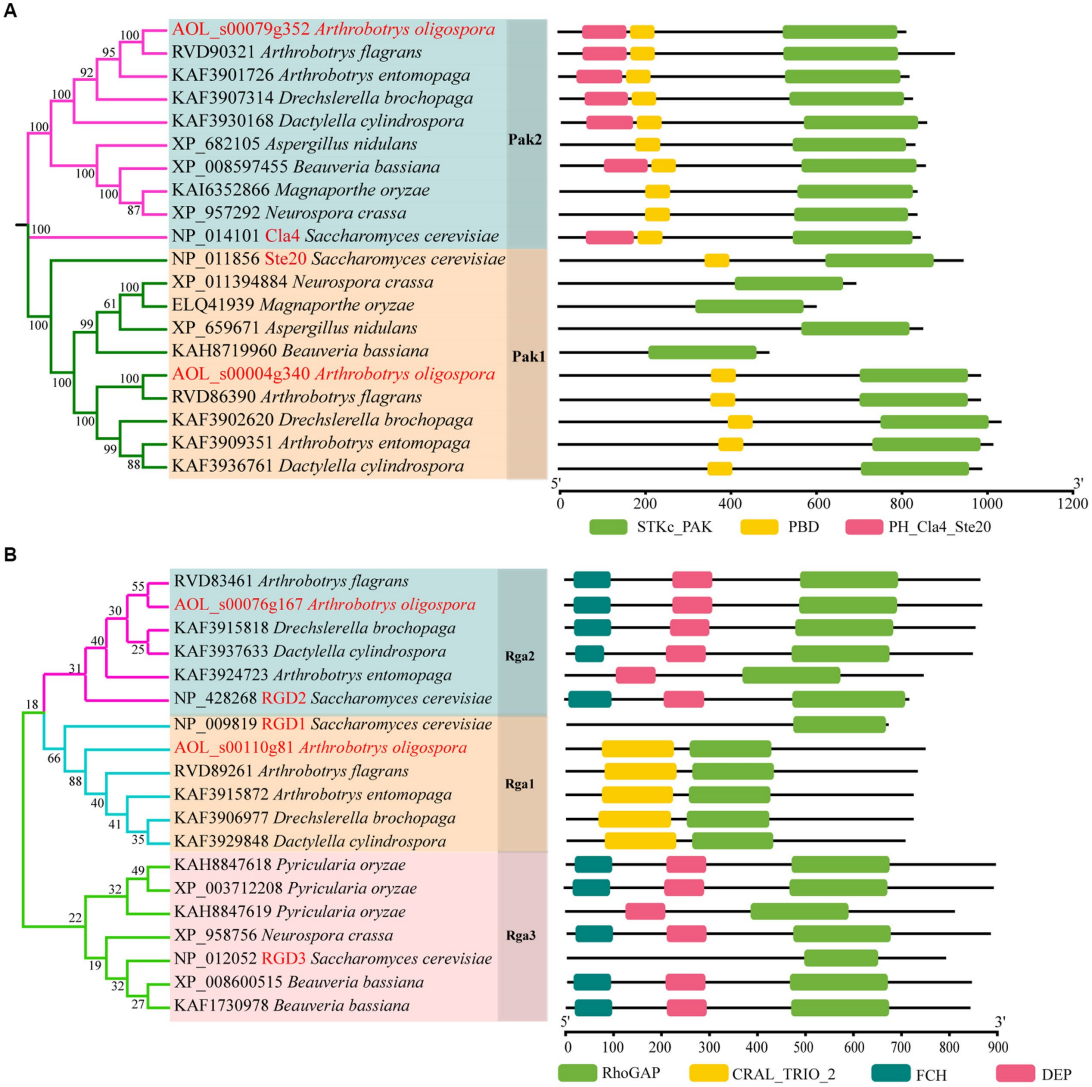
Phylogenetic analysis of Rho-GAPs and PAKs. **(A, B)** Phylogenetic and structure domain analyses of Rho-GAP and PAK orthologs from different fungi.

Based on the orthologous proteins in *S. cerevisiae*, we classified PAKs into Pak1 and Pak2, and Rho-GAP into Rga1, Rga2 and Rga3 (Figure 1). And two PAKs (AoPak1, AOL_s00004g340; AoPak2, AOL_s00079g352) and two Rho-GAPs (AoRga1, AOL_s00110g81; AoRga2, AOL s00076g167) were identified in *A. oligospora*. Bioinformatic analyses showed that AoPAKs were conserved between *S. cerevisiae* and filamentous fungi, and especially in five NT fungi, including *A. flagrans*, *D. cylindrospora*, *A. entomopaga*, *D. brochopaga*, and *A. oligospora*, wherein AoPak1 and AoPak2 showed high similarity (71.5–96.0% and 70.0–94.2%) with the orthologs from the other four NT fungi, respectively (Supplementary Table S2). Phylogenetic and domain analyses showed that *A. flagrans* is the closest evolutionary relative to *A. oligospora*, and the PAKs contain an STKc_PAK and a PBD domain in five NT fungi. In addition, AoPak2 also contains a PH_Cla4_Ste20 domain, and AoPak2 has a higher homology with Cla4 of *S. cerevisiae*, while AoPak1 is more similar to Ste20 (Figure 1A). Rho-GAPs contain a conserved Rho-GAP domain, and AoRga1 is conserved only in NT fungi with a high homology of 71.9–92.5% (Figure 1B; Supplementary Table S3).

### 3.2. *Aopak1* and *Aorga1* have no significant roles on the mycelial growth

**Figure 2:**
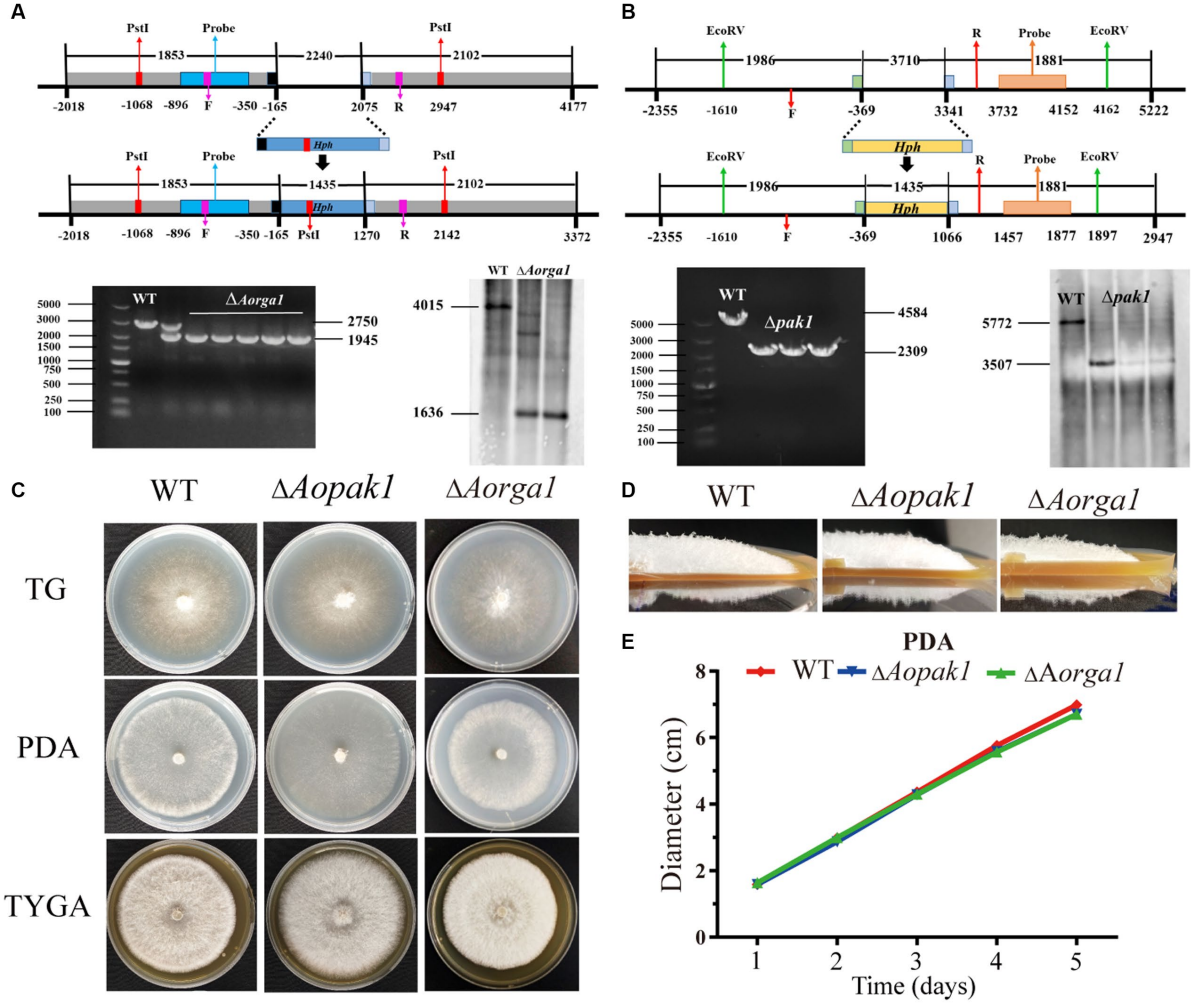
Comparison of the mycelial growth between WT and mutants (∆*Aopak1* and ∆*Aorga1*). **(A, B)** Validation of *Aorga1* and *Aopak1* knockout strains using PCR and Southern blot. F, 5′ primer for validation of the transformants by PCR. R, 3′ primer for validation of the transformants by PCR. **(C)** Colony morphology of fungal strains cultured on TG, PDA and TYGA media for 5 days at 28°C. **(D)** The morphology of aerial hyphae incubated on TGYA medium for 5 days. **(E)** Comparison of mycelial growth on PDA medium.

To determine the functions of *Aopaks* and *Aorga1* in *A. oligospora*, we tried to delete the genes of *Aopak1*, *Aopak2* and *Aorga1* via homologous recombination. Finally, we successfully obtained ∆*Aopak1* and ∆*Aorga1* mutants with more than three positive transformants for each gene. The transformants were identified by genomic PCR amplification and Southern blotting analysis (Figures 2A,B). Because the individual transformants of each gene showed similar phenotypic traits, a single transformant for each gene was randomly selected for subsequent analysis.

WT and mutant strains were cultured on TG, PDA, and TYGA media for 5 days, and the colony morphology and mycelial growth between the WT and two mutant strains showed no obvious differences (Figure 2; Supplementary Figure S1). The mean colony diameters of the WT, ∆*Aopak1* and ∆*Aorga1* mutant strains cultured on TG media for 5 days were 6.65, 7.02, and 6.93 cm, respectively; these measurements were 6.98, 6.70, and 6.70 cm on PDA and 6.77, 7.05, and 6.75 cm on TYGA.

### 3.3. *Aopak1* and *Aorga1* contribute to trap formation and extracellular proteolytic activity

**Figure 3:**
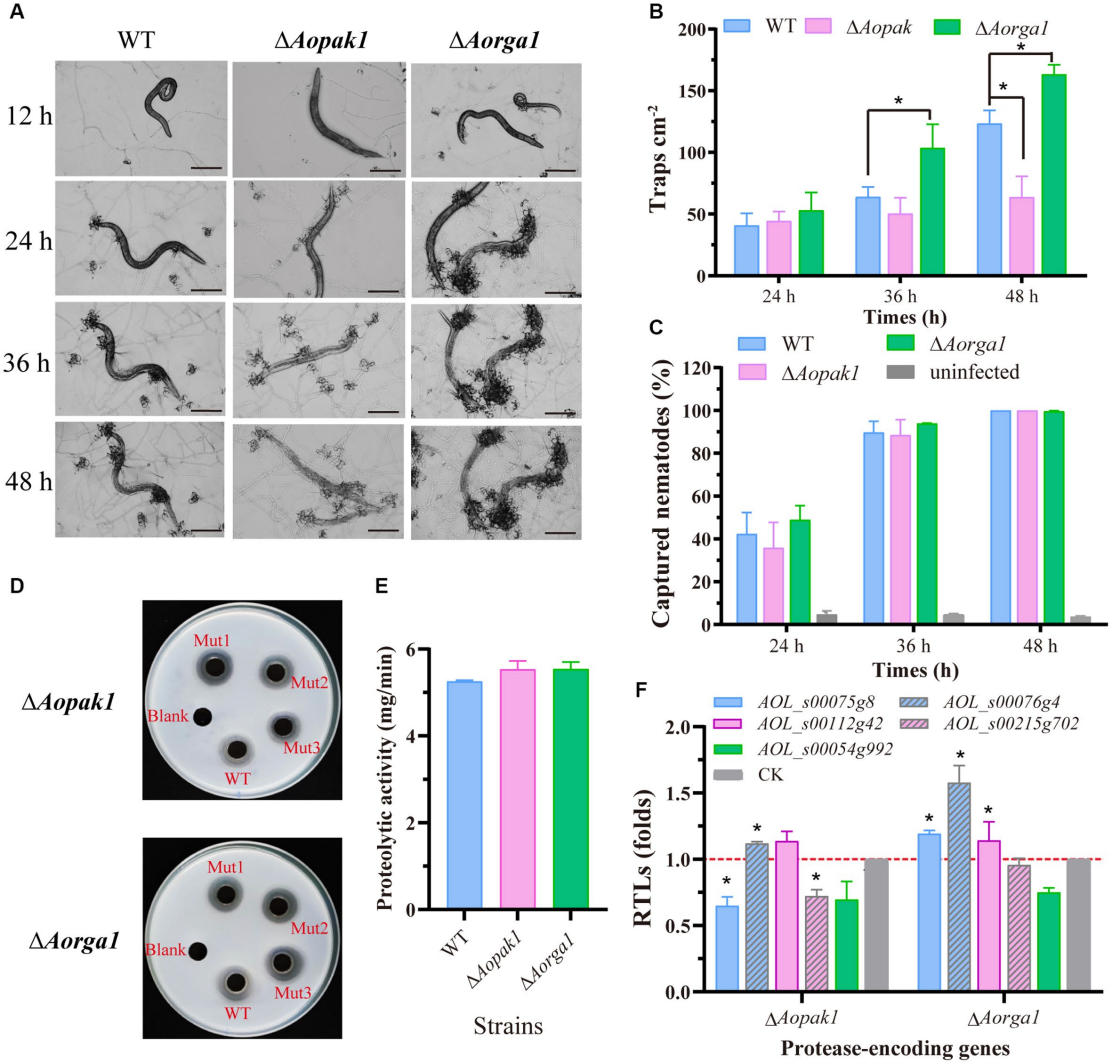
Comparison of trap formation and extracellular proteolytic activity in the WT and mutant strains. **(A)** The morphology of captured nematodes and traps at 12, 24, 36, and 48 h post-induction (hpi). Bar = 100 μm. **(B)** Number of traps at three different time points (24, 36, and 48 hpi). *, *p* < 0.05. **(C)** Comparison of captured nematodes in WT and mutants (∆*Aopak1* and ∆*Aorga1*). **(D, E)** Qualitative and quantitative determination of extracellular proteolytic activity. **(F)** Relative transcript levels (RTLs) of protease-related genes between the WT strain and mutants (Δ*Aopak1* and Δ*Aorga1*) cultured on PDA medium for 5 days, and then inducted by *C. elegans* for 24 h. * *p* < 0.05.

We added 200 nematodes to each plate of WT, ∆*Aopak1*, and ∆*Aorga1* mutant strains to induce trap formation. The WT and mutants produced traps at 12 h post-induction (hpi), but there were differences in the ability of trap formation (Figure 3A). Compared with WT, the trap numbers of ∆*Aorga1* mutants were significantly increased at 36 and 48 hpi, whereas those of ∆*Aopak1* were decreased, most notably at 48 hpi (Figure 3B). However, the nematode predatory efficiency for Δ*Aopak1* and ∆*Aorga1* was not significantly different from that of the WT at different time points (Figure 3C).

Furthermore, the disruption of the *Aopak1* and *Aorga1* genes resulted in a slight increase in proteolytic activity compared to WT strains (Figures 3D,E), which is consistent with the nematode-digesting capacity of the strains. To further explore the regulation of *Aopak1* and *Aorga1* in terms of serine proteases, the relative transcript levels (RTLs) of five protease-related genes were determined by RT-PCR in WT, ∆*Aopak1*, and ∆*Aorga1* strains. The RTL of *76 g4* was significantly up-regulated (*p* < 0.05) in both ∆*Aopak1* and ∆*Aorga1* mutants, and the RTL of *54 g992* was significantly down-regulated (*p* < 0.05) in the *Aopak1* and *Aorga1* disruption strains. The opposite expression trend of *75 g8* was observed in the ∆*Aopak1* and ∆*Aorga1* mutants (Figure 3F).

### 3.4. *Aopak1* and *Aorga1* play a crucial role in sporulation

**Figure 4:**
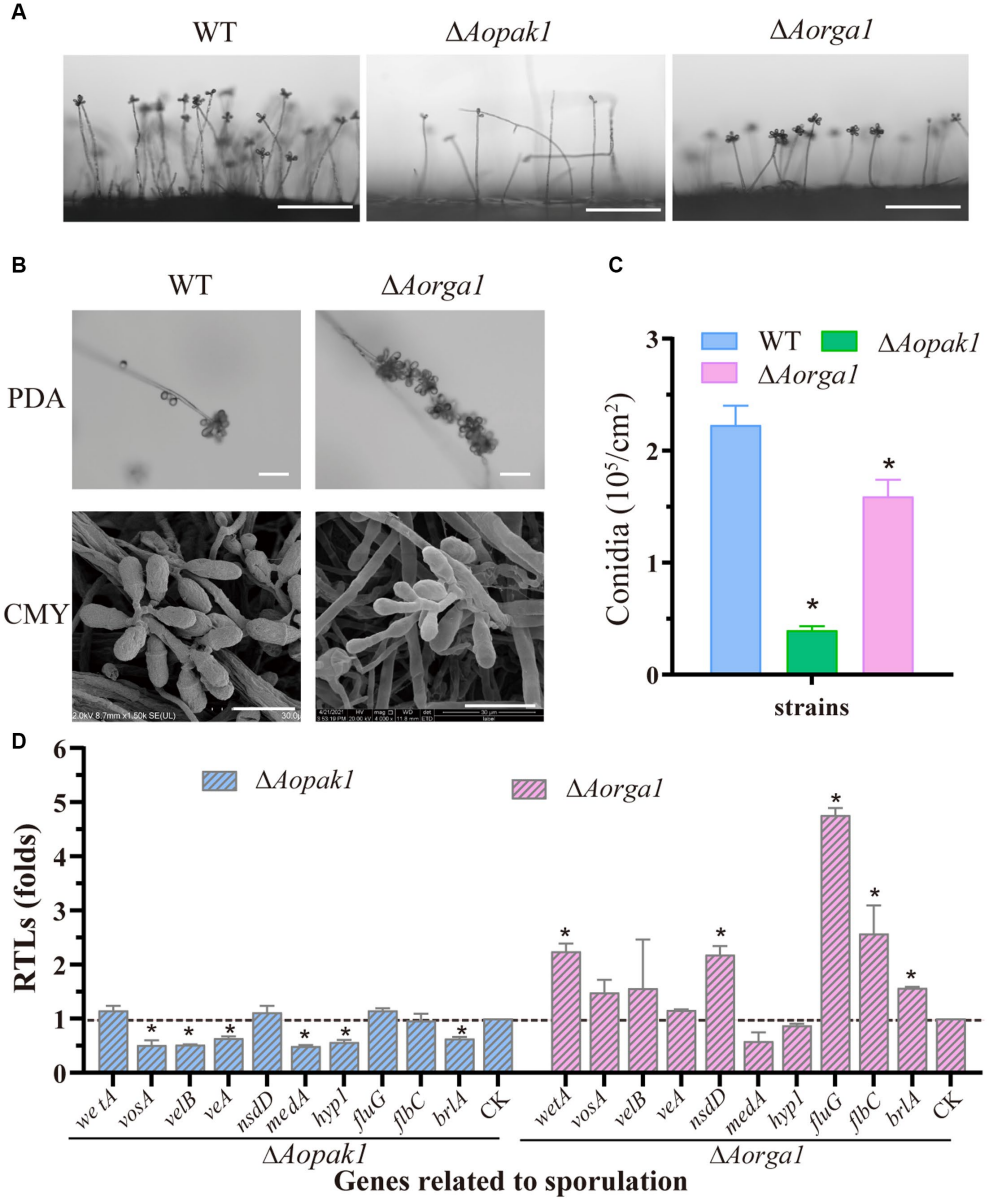
Comparison of conidiation and the transcript level analysis of sporulation-related genes between the WT and mutant strains. **(A)** Conidiophore observation in WT and mutants (Δ*Aopak1* and Δ*Aorga1*) cultured on PDA for 3 days. Bar = 100 μm. **(B)** Comparison of conidiophore morphology between the WT and Δ*Aorga1* mutant strains cultured on CMY for 7 days. Bar = 20 μm. **(C)** Spore yield statistics for WT and mutants cultured on CMY for 15 days. * *p* < 0.05. **(D)** Relative transcription levels (RTLs) of sporulation-related genes between the WT and mutant strains. The expression of the corresponding genes in WT was normalized and used as CK. * *p* < 0.05.

The deletion of both *Aopak1* and *Aorga1* resulted in a remarkable reduction in conidiophores. Particularly in the Δ*Aopak1* mutants, not only did the conidiophores become more sparse, but the number of spores on each conidiophore was also remarkably decreased (Figure 4A). On the contrary, the conidia attached on the conidiophores of the Δ*Aorga1* mutant strains showed multiple whorls (Figure 4B). As a result, the spore yields of the mutants were significantly reduced compared to the WT, and the conidia yields of WT, Δ*Aopak1*, and Δ*Aorga1* strains were 2.23, 0.40, and 1.59 × 10^5^ conidia/cm^2^, respectively (Figure 4C). Following this, the transcriptional levels of most sporulation-related genes were down-regulated in the Δ*Aopak1* mutant, especially *AovosA*, *AovelB*, *AoveA*, *AomedA*, *Aohyp1*, and *AobrlA*. However, these genes were up-regulated in the Δ*Aorga1* mutant, except for *AomedA* and *Aohyp1* (Figure 4D).

### 3.5. *Aopak1* and *Aorga1* regulate stress resistance and the number of nuclei

**Figure 5:**
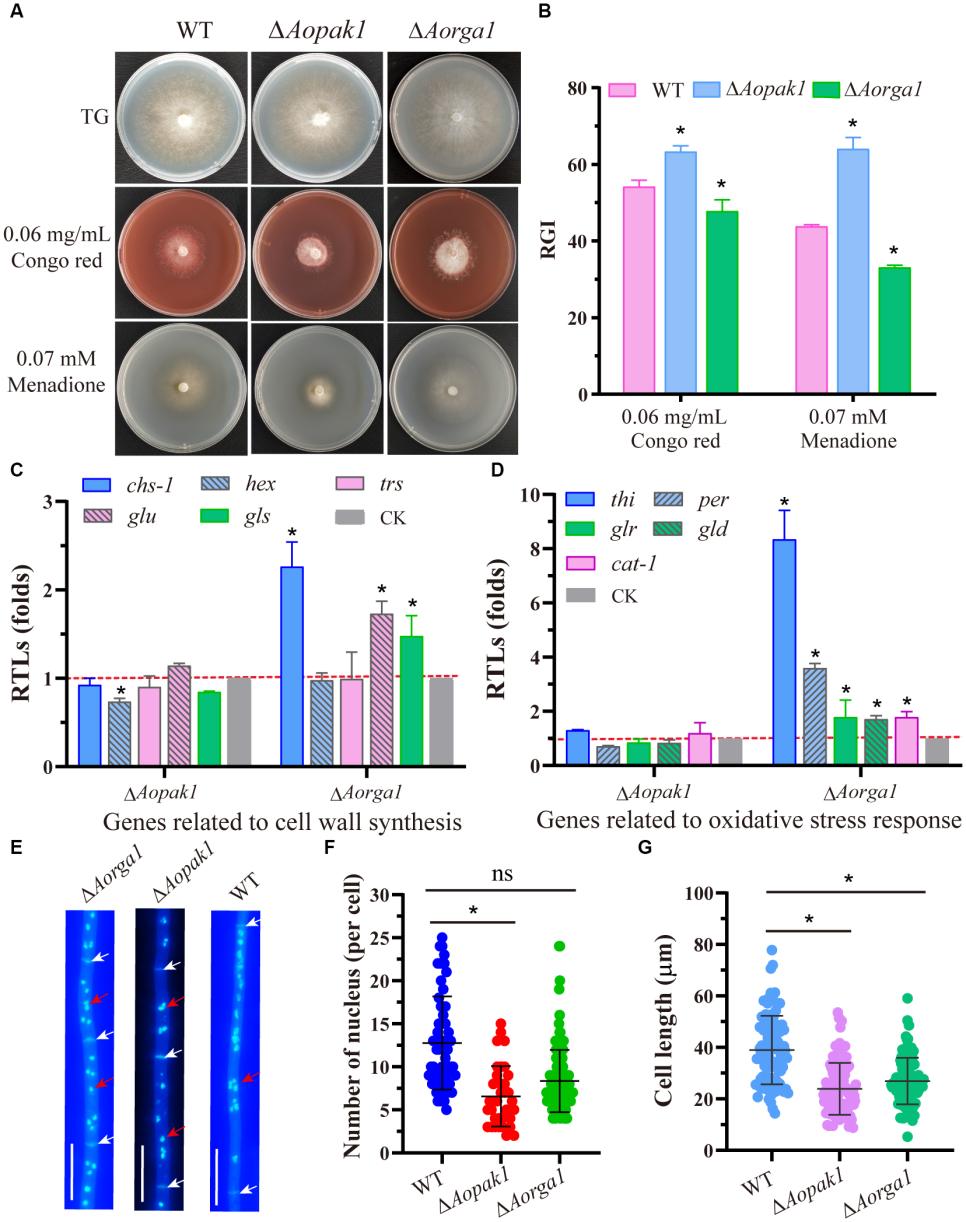
Comparison of stress responses and the numbers of nuclei. **(A)** Colony morphology of WT and mutants cultured on TG plates supplemented with 0.06 mg/mL Congo red and 0.07 mM menadione, respectively. **(B)** Relative growth inhibition (RGI) rate of WT and mutants cultured on **(A)**. * *p* < 0.05. **(C, D)** Relative transcription levels (RTLs) of genes associated with cell wall synthesis and oxidative stress response in the mutants compared with the WT strain. * *p* < 0.05. **(E)** Representative images of nuclei stained with DAPI and visualized using a fluorescent microscope. White arrow, septum. Red arrow, nuclei. Bar = 10 μm. **(F)** Statistical analysis of the number of nuclei. ns, not statistically significant, * *p* < 0.05. **(G)** Statistical analysis of cell length. ns, not statistically significant, * *p* < 0.05.

To probe the roles of *Aopak1* and *Aorga1* in stress response, we treated strains with a cell-wall-disturbing reagent (Congo red) and oxidant (menadione). The results show that the Δ*Aopak1* mutant was more sensitive to the cell wall and oxidative stress reagents, as the RGI value of the Δ*Aopak1* mutant was significantly higher than that of the WT strain. However, the sensitivity of the Δ*Aorga1* mutant to the cell wall and oxidative stress reagents was decreased, and the RGI values here were significantly lower than those of WT (Figures 5A,B). *Aohex* (coding hexokinase), a gene related to cell wall biosynthesis, was significantly down-regulated in the Δ*Aopak1* mutant, whereas the RTLs of three cell-wall-synthesis-related genes (*Aochs-1*, coding chitin synthases; *Aoglu*, coding *β*-glucosidase; and *Aogls*, coding 1,3-*β*-glucan synthase) were remarkably up-regulated in the Δ*Aorga1* mutant (Figure 5C). Similarly, five oxidative-stress-response-related genes, including *Aothi* (coding thioredoxin), *Aoper* (coding peroxidase), *Aoglr* (glutathione reductase), *Aogld* (coding glutathione dehydrogenase), and *Aocat-1* (coding catalase), were significantly up-regulated in the Δ*Aorga1* mutant (Figure 5D).

Furthermore, disrupting *Aopak1* and *Aorga1* resulted in a decrease in the nuclei number of per cell and cell length. Statistical analysis showed that each cell has an average of 13, 7, and 8 nuclei in the WT, Δ*Aopak1*, and Δ*Aorga1* mutants, respectively (Figures 5E,F). The mean cell length of Δ*Aopak1* and Δ*Aorga1* mutants was significantly shorter than that of WT (Figure 5G).

### 3.6. AoRga1 indirectly regulates AoPak1 by regulating small GTPases

**Figure 6:**
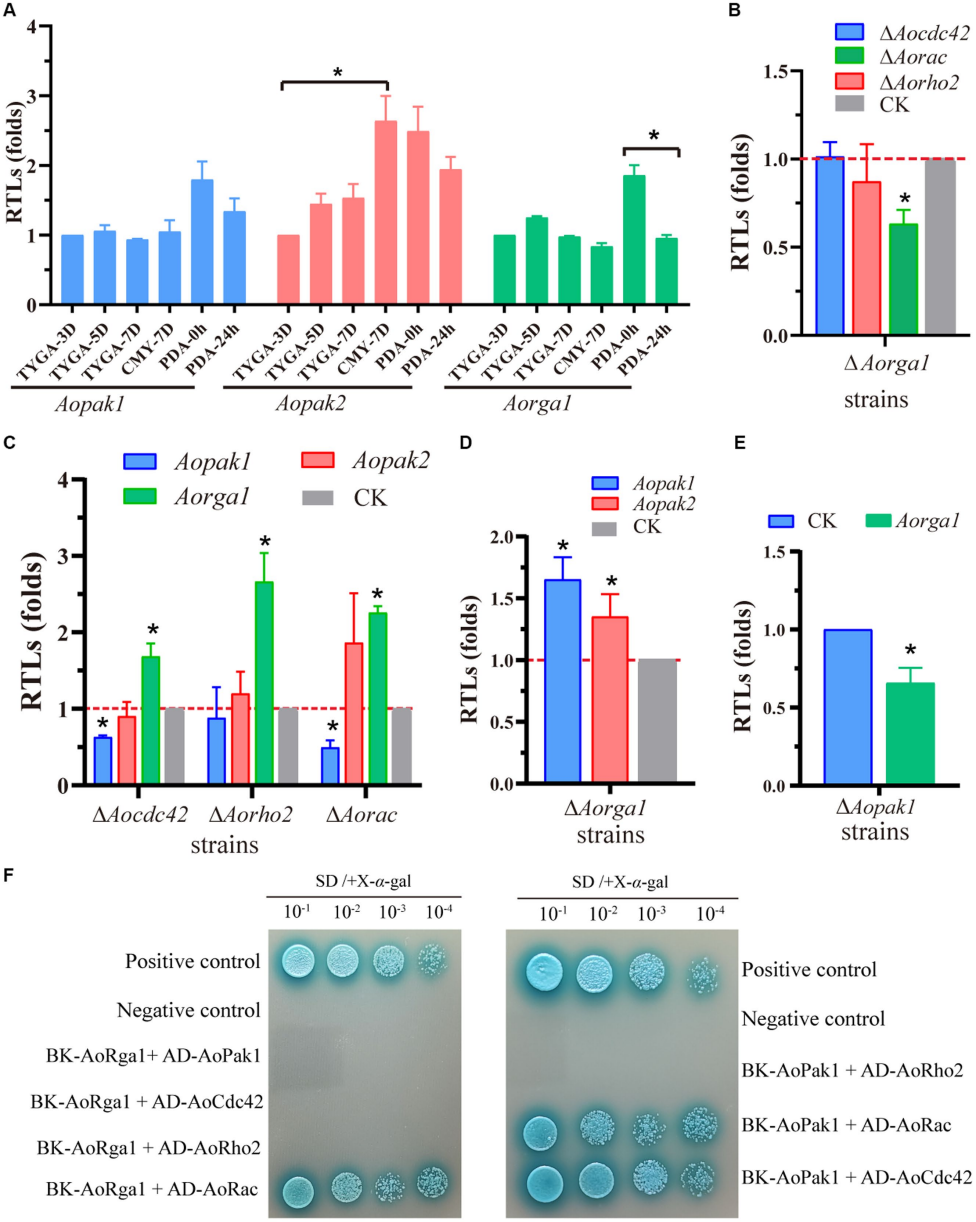
AoRho-GAP indirectly regulates AoPAKs by regulating Rho GTPases. **(A)** Transcription patterns of *Aopak1*, *Aopak2*, and *Aorga1* in different developmental stages of WT. * *p* < 0.05. **(B–E)** Relative transcription levels (RTLs) of *Aopak1*, *Aopak2*, *Aorga1*, *Aocdc42*, *Aorho2*, and *Aorac* in different mutants. The expression of the corresponding genes in WT was normalized and used as CK. * *p* < 0.05. **(F)** Verification of the interaction relationship between AoPak1, AoRga1, and Rho GTPases (AoCdc42, AoRho2, and AoRac) by Y2H assay. The interaction of pGBKT7-53 with pGADT7-T was used as a positive control, and the interaction of pGBKT7-lam with ADT7-T was used as a negative control.

To probe the regulation mechanism of *Aopak* and *Aorga1* in *A. oligospora*, we determined the transcription patterns of three genes at different developmental stages. The transcription level of *Aopak2* in the sporulation stage was significantly higher than that in the vegetative growth stage, and the transcriptional level of *Aorga1* was remarkably down-regulated at 24 h after the induction of *C. elegans* (Figure 6A). Previous studies showed that small GTPases can bind to the CRIB domain of PAKs and are regulated by Rho-GAPs (Boyce and Andrianopoulos, 2011; Ye et al., 2014). Therefore, we detected the transcription of three genes encoding the Rho GTPases in Δ*Aorga1* mutants, and we found that *Aorac* was significantly down-regulated (Figure 6B). Moreover, the *Aorga1* was significantly up-regulated in Δ*Aocdc42*, Δ*Aorho2*, and Δ*Aorac* mutants, whereas *Aopak1* was down-regulated in three mutants (Figure 6C). Interestingly, *Aorga1* was down-regulated in Δ*Aopak1* mutants, while *Aopak1* and *Aopak2* were both up-regulated in Δ*Aorga1* mutants (Figures 6D,E). In addition, Y2H analysis showed that AoPak1 cannot interact with AoRga1, but they can both interact with AoRac, and AoPak1 can also interact with AoCdc42 (Figure 6F; Supplementary Figure S2). These results demonstrate that AoRga1 indirectly regulates AoPAK by regulating smallGTPases.

## 4. Discussion

Signaling pathways play a crucial role in the vegetative growth and development in fungi, as they can sense alterations in various physical and chemical stimuli in the environment and translate them into intracellular signals (Yang et al., 2011; Zhu et al., 2022a). PAKs function upstream of the MAPK cascades and are regulated by small GTPases, which are negatively regulated by Rho-GAPs (Dan et al., 2001; Boyce and Andrianopoulos, 2011; Rawat and Chernoff, 2015). PAKs participate in multiple phenotypes in fungi, including polarized morphogenesis (Leveleki et al., 2004; Nichols et al., 2004), cell morphogenesis (Boyce et al., 2009), conidial germination (Boyce and Andrianopoulos, 2007), virulence and hyphal formation (Leberer et al., 1997), and pathogenicity (Li et al., 2004; Rolke and Tudzynski, 2008). In addition, Rho-GAPs play a vital role in cell proliferation (Shang et al., 2007), pathogenicity (Ye et al., 2014), and growth (Yang et al., 2003). Here, we found that AoPak1 and AoRga1 were also involved in multiple biological processes in the NT fungus *A. oligospora*, such as sporulation, trap formation, and stress response.

The disruption of *Aopak1* or *Aorga1* had no effect on hyphal growth (Figure 2). Similarly, the deletion of *skm1* (coding a PAK protein) manifested no detectable phenotype under laboratory conditions, but the overexpression of *skm1*, *ste20* or *cla4* lacking an N-terminus led to growth arrest in *S. cerevisiae* (Martin et al., 1997). However, the deletion of *Molrg1* resulted in a dramatic decrease in the growth rate of aerial hyphae in *M. oryzae*, while the six other Rho-GAP-domain containing genes did not impact vegetative growth due to functional redundancy (Ye et al., 2014). In *M. oryzae*, ∆*mst20* mutants were reduced during aerial hyphae growth (Li et al., 2004). The presence of ∆*cla4* mutants completely removed the ability to form filaments (Szabo, 2001). Based on these results we speculate functional redundancy between *Aopak1* and *Aopak2*, meaning the deletion of *Aopak1* does not affect mycelial growth.

The traps are important infectious structures of NT fungi, and mature trap formation is essential to their pathogenicity (Zhu et al., 2022a). The disruption of *Aopak1* resulted in a reduction in trap number at 48 hpi, while the numbers of traps were increased in ∆*Aorga1* mutants at 36 and 48 hpi (Figure 3B). However, the nematode predation abilities of Δ*Aopak1* and Δ*Aorga1* mutants were similar to that of WT (Figure 3C). NT fungi can secrete cuticle-degrading serine proteases, which act as key mediators of virulence against nematodes, and many related gens have been cloned (Yang et al., 2005, 2017; Liang et al., 2011; Tzean et al., 2016). For example, serine protease PII was first identified in *A. oligospora*; it can immobilize the free-living nematode *Panagrellus redivivus*, and degrade the nematode cuticle (Tunlid et al., 1994). Herein, we analyzed the expressions of serine protease-related genes and found that most genes were differently expressed in Δ*Aopak1* and Δ*Aorga1* mutants, and *76 g4* (encoding cuticle-degrading protease PII) was remarkably up-regulated in Δ*Aopak1* and Δ*Aorga1* mutants, suggesting that *Aopak1* and *Aorga1* play crucial roles in the regulation of extracellular proteolytic activity. In addition, previous studies have showed that *cla4* is required for pathogenesis in *C. albicans* (Leberer et al., 1997), *B. maydis* (Kitade et al., 2019), *U. maydis* (Leveleki et al., 2004), *C. purpurea* (Rolke and Tudzynski, 2008), and *V. dahliae* (Tian et al., 2015). Meanwhile, *mst20* plays a key role in the pathogenicity of *M. grisea* (Li et al., 2004), *U. maydis* (Smith et al., 2004), and *C. neoformans* (Nichols et al., 2004), but has a negligible effect on pathogenicity in *B. maydis* (Kitade et al., 2019). Moreover, the deletion of *Molrg1* resulted in a complete loss of pathogenicity in *M. oryzae*, but the appressorial formation and pathogenicity of six genes’ (coding Rho-GAP proteins) mutants were similar to those of WT (Ye et al., 2014). These results indicate that the roles of PAKs and Rho-GAPs in pathogenicity vary among fungal species, and there is functional redundancy among homologous genes. Similarly, *Aopak* and *Aorga1* have been shown to be involved in trap formation in *A. oligospora*, and *Aopak2* may complement the functional defects caused by the knockout of *Aopak1*. In contrast to studies in other species, the knockout of *Aorga1* here resulted in an increase in the number of traps and an accelerated rate of nematode digestion. The specific mechanisms involved here need to be further explored.

The main component of biocontrol agents is conidia, and the ability to produce spores is one of the key factors at play in the fecundity and fitness of biocontrol fungi (Zhang et al., 2019). Studies have shown that the deletion of *Morga1* results in a high percentage of larger or gherkin-shaped conidia and decreases in conidiation (Ye et al., 2014). In *M. grisea*, conidiation was reduced in both ∆*chm1* and ∆*mst20* mutants, most notably in the former, which showed more conidia with abnormal morphologies (Li et al., 2004). The conidiation of ∆*ste20* mutants was similar to that of WT, while ∆*cla4* strains showed more severe defects than the WT strain in *Bipolaris maydis* (Kitade et al., 2019). Consistently with the results for *M. grisea*, the deletion of *Aopak1* and *Aorga1* resulted in a significantly decrease in spore yield (Figure 4C), and the conidiophore morphology of Δ*Aorga1* mutants was abnormal (Figure 4B). The transcriptions of several sporulation-related genes (Bai et al., 2023b), including *AovosA*, *AovelB*, *AoveA*, *AomedA*, *Aohyp1*, and *AobrlA*, were significantly down-regulated in the Δ*Aopak1* mutant, while *AowetA*, *AonsdD*, *AofluG*, *AoflbC*, and *AobrlA* were up-regulated in the Δ*Aorga1* mutant (Figure 4D). Except for *brlA*, the differentially expressed genes regulated by *Aopak1* and *Aorga1* were different, and these genes were regulated in opposite patterns in the Δ*Aopak1* and Δ*Aorga1* mutants, suggesting that AoPak1 and AoRga1 play different roles in the regulation of conidia formation.

Stress response is essential to fungi’s sense of their environment, and enables them to make timely adjustments to adapt to changes. In this study, we found that the deletion of *Aopak1* led to increased sensitivity to oxidant and cell-wall-disturbing reagents, whereas the disruption of *Aorga1* resulted in an increased resistance to these stressors (Figure 5B). The alterations in the transcriptional levels of related genes were consistent with the corresponding phenotypes, especially in the Δ*Aorga1* mutant, where most of the related genes associated with cell wall synthesis and oxidative stress response were significantly up-regulated (Figures 4C,D). These results suggest that *Aopak1* and *Aorga1* play critical and diverse roles in stress response. Previous studies have shown that PAKs, upstream of MAPK cascades, are indirectly regulated by Rho-GAPs (Bassilana et al., 2005; Boyce and Andrianopoulos, 2011; Ye et al., 2014). In *S. cerevisiae*, activated Ste20 phosphorylates the MAPK kinase. The kinase Ste11 can activate the MAPK pathways regulating cell wall integrity and filamentous growth (Wu et al., 1995; van Drogen et al., 2000). In *A. oligospora*, Bck1, Mkk1, and Slt2 signaling cascade has been proved to be involved in multi-stress tolerance (Zhen et al., 2018; Xie et al., 2021). In addition, some transcription factors downstream of MAPK cascades have been confirmed to be involved in stress resistance, such as Ste12 (Bai et al., 2023a), RlmA (Yang et al., 2023), and Swi6 (Xie et al., 2022). Therefore, AoPaks and AoRho-GAPs may influence the stress response by regulating the downstream effectors, including the MAPK cascades and related transcription factors.

**Figure 7:**
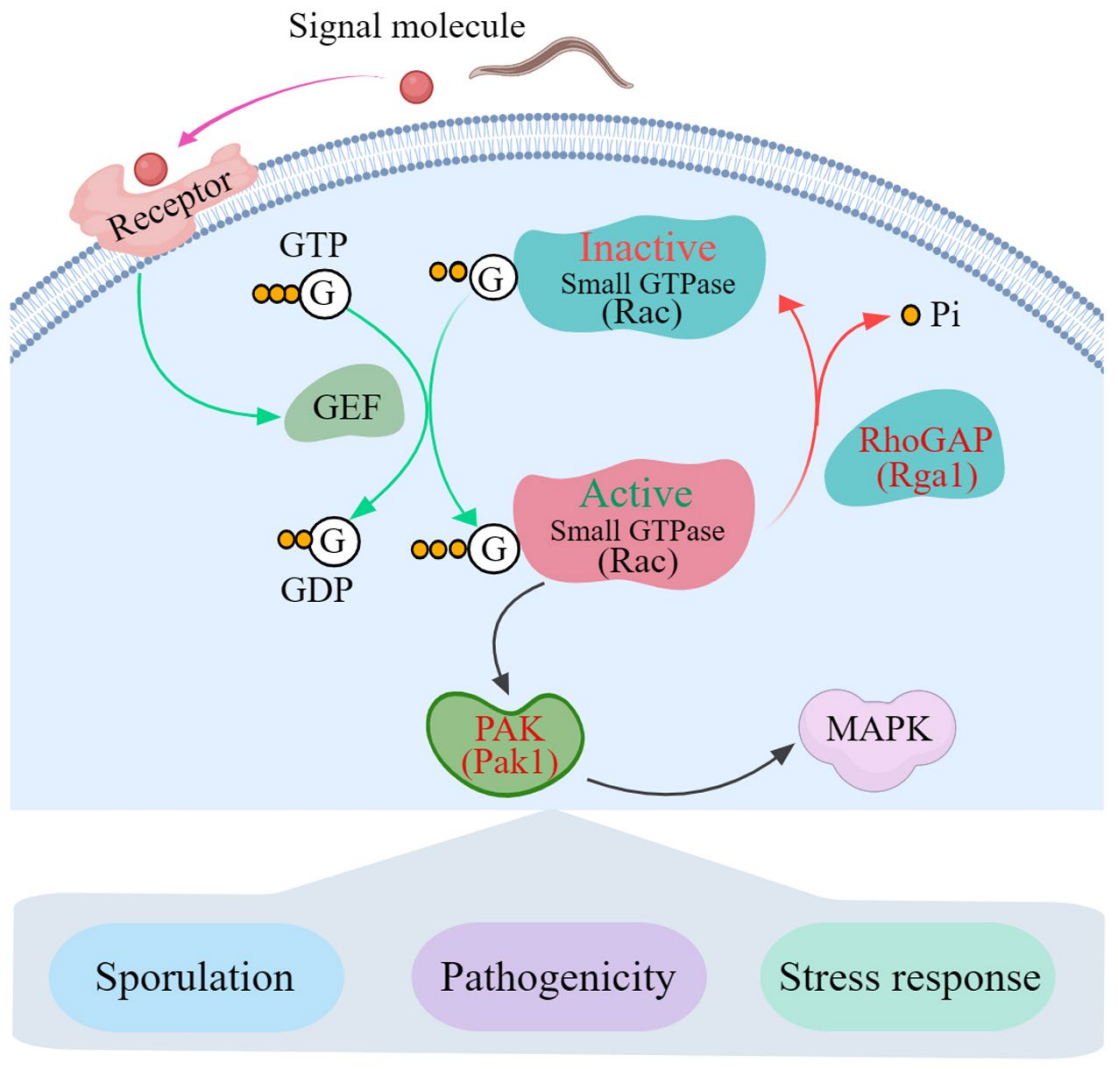
A proposed interaction model between Rho-GAP and PAKs in *A. oligospora*. GTP, Guanosine triphosphate; GDP, Guanosine-5′-diphosphate; GEF, guanine-nucleotide exchange factor; RhoGAP, Rho GTPase-activating protein; PAK, p21-GTPase-activated protein kinase; MAPK, mitogen-activated protein kinase.

The transcription pattern analysis and Y2H results show that Rho GTPases link AoPAK and AoRho-GAP, enabling regulation between them, and there is negative feedback between *Aopak* and *Aorga1* (Figure 6). Studies have confirmed that small GTPases are negatively regulated by Rho-GAP (Etienne-Manneville and Hall, 2002; Burridge and Wennerberg, 2004). In this study, *Aopak1* was shown to be positively regulated by the phenotype of *A. oligospora*, similarly to the effect seen in other species, such as *C. albicans* (Leberer et al., 1997), *B. maydis* (Kitade et al., 2019), *U. maydis* (Leveleki et al., 2004), *C. purpurea* (Rolke and Tudzynski, 2008), *V. dahliae* (Tian et al., 2015), *M. grisea* (Li et al., 2004), and *C. neoformans* (Nichols et al., 2004). On the other hand, the deletion of *Aorga1* resulted in alterations in the number of traps, nematode digestion efficiency, and stress response, which is a novel finding compared to other species. This may be related to the homology of the protein sequence in filamentous fungi. These results can be partly explained by alterations in the transcription pattern of the related genes in each mutant strain, and deserve more detailed study. Combined with the results of this study, we see that AoPAKs regulate the sporulation, trap formation, stress resistance, and number of nuclei via the indirect regulation of AoRho-GAP in *A. oligospora* (Figure 7). Our study elucidates the mechanisms involved in signal transduction pathways regulating conidia and trap formation, and it highlights the roles played by AoPAKs and AoRho-GAPs in improving the qualities of biocontrol fungi.

## 5. Conclusion

We identified and characterized a Rho GAP and two PAK-coding genes, *Aorga1*, *Aopak1* and *Aopak2*, from the NT fungus *A. oligospora*. Our results show that *Aopak1* and *Aorga1* play crucial roles in conidiation, trap formation, and response to oxidant and cell-wall-disturbing reagents. In particular, *Aorga1* negatively regulates trap formation and nematode digestion, which is a novel finding in the context of other fungi. Our findings provide new insights into the Rho-GAP- and PAKs-mediated signaling pathways that regulate trap formation, conidiation, and stress resistance in NT fungi.

## Data availability statement

The original contributions presented in the study are included in the article/supplementary material, further inquiries can be directed to the corresponding author.

## Author contributions

JY conceived and designed the study, and revised the manuscript. MZ wrote the manuscript. MZ and YL conducted the experiments. XY, LZ, YS, and SD analyzed the data. All authors contributed to the article and approved the submitted version.

## Footnotes

^1^ http://pfam-legacy.xfam.org/
