# Supplementary material for: p21-activated kinase is involved in the sporulation, pathogenicity, and stress response of Arthrobotrys oligospora under the indirect regulation of Rho GTPase-activating protein
Source: Front Microbiol. 2023 Sep 14;14:1235283. doi: 10.3389/fmicb.2023.1235283 (PMC10537225; doi:10.3389/fmicb.2023.1235283)
Supplement: Supplementary file 1 [file Data_Sheet_1.docx]

**Supplementary figures**

**Figure S1 Comparison of mycelial growth of the WT and mutant strains on TG and TYGA media.**


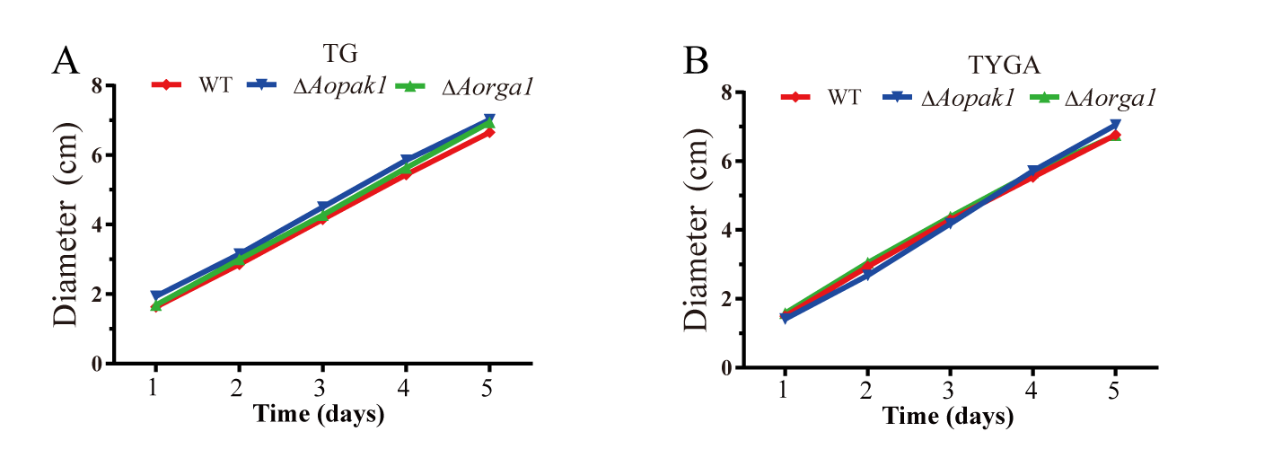


**Figure S2 Y2H assay to detect the self-activating activities of AoPak1, AoRga1, AoCdc42, AoRho2, and AoRac.**


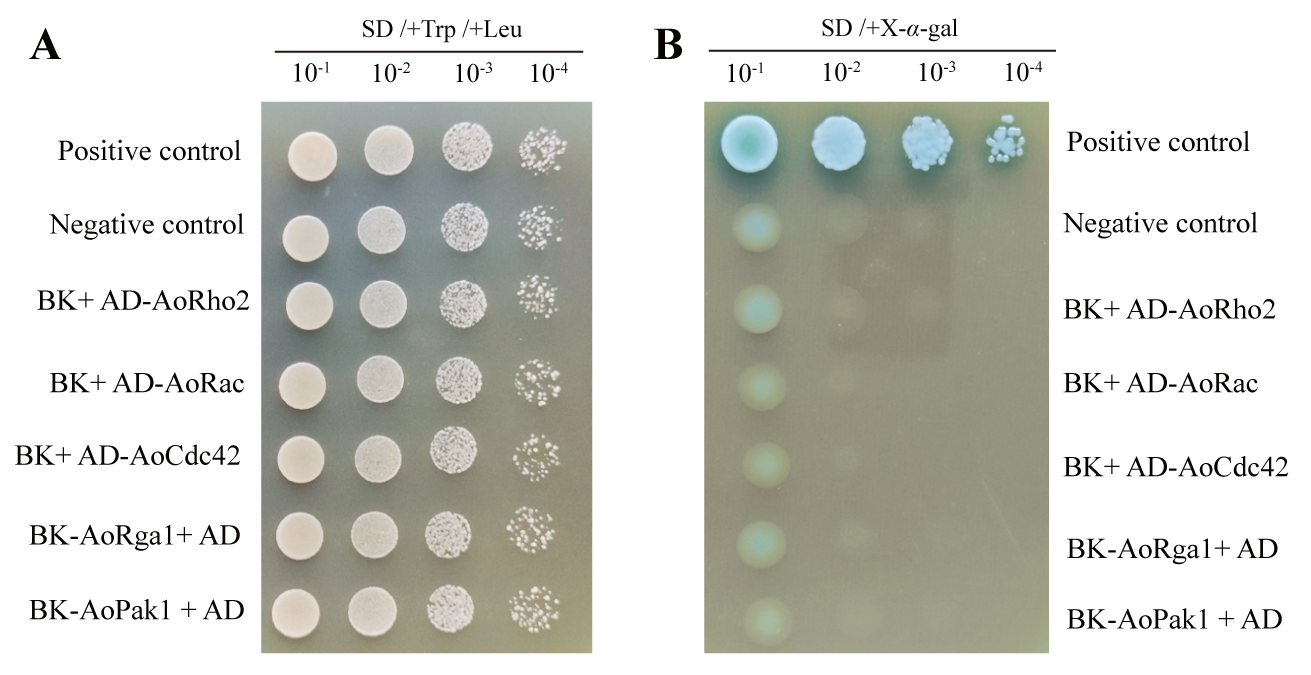


**Supplementary tables**

| **Table S1.** **The mutant strains used in this study.**   \| Gene names \| Gene ID \| Replacement segments \| \| --- \| --- \| --- \| \| *Aocdc42* \| *AOL_s00043g439* \| refer to (Yang et al., 2022) \| \| *Aorac* \| *AOL_s00079g171* \| refer to (Yang et al., 2022) \| \| *Aorho2* \| *AOL_s00215g387* \| refer to (Yang et al., 2022) \| \| *Aopak1* \| *AOL_s00004g340* \| Shown in Figure 2B \| \| *Aorga1* \| *AOL_s00110g81* \| Shown in Figure 2A \|   **Table S2. The partial properties of orthologous PAKs.** The sequence similarity of AoPAKs with other orthologs from different fungi was analyzed using Geneious 4.8.5 software.   \| Fungal strain and gene ID \| Similarity \| Molecular weight \| Isoelectric point \| \| --- \| --- \| --- \| --- \| \| *Arthrobotrys oligospora* AOL_s00004g340 \| 100% (AoPak1) \| 107.746 kDa \| 10.1 \| \| *Arthrobotrys entomopaga* KAF3909351 \| 74.6% (AoPak1) \| 110.900 kDa \| 10.14 \| \| *Arthrobotrys flagrans* RVD86390 \| 96% (AoPak1) \| 107.707 kDa \| 10.17 \| \| *Aspergillus nidulans* XP_659671 \| 51% (AoPak1) \| 93.421 kDa \| 9.84 \| \| *Beauveria bassiana* KAH8719960 \| 74% (AoPak1) \| 53.615 kDa \| 9.39 \| \| *Dactylella cylindrospora* KAF3936761 \| 71.6% (AoPak1) \| 107.996 kDa \| 10.02 \| \| *Drechslerella brochopaga* KAF3902620 \| 71.5% (AoPak1) \| 112.628 kDa \| 10.06 \| \| *Neurospora crassa* XP_011394884 \| 64.2% (AoPak1) \| 76.060 kDa \| 9.67 \| \| *Magnaporthe oryzae* ELQ41939 \| 68.3% (AoPak1) \| 66.933 kDa \| 9.05 \| \| *Saccharomyces cerevisiae* NP_011856 \| 38.8% (AoPak1) \| 102.362 kDa \| 7.36 \| \| *Arthrobotrys oligospora* AOL_s00079g352 \| 100% (AoPak2) \| 90.369 kDa \| 8.82 \| \| *Aspergillus nidulans* XP_682105 \| 47.7% (AoPak2) \| 90.908 kDa \| 9.26 \| \| *Beauveria bassiana* XP_008597455 \| 50.9% (AoPak2) \| 93.216 kDa \| 9.69 \| \| *Dactylella cylindrospora* KAF3930168 \| 70% (AoPak2) \| 95.160 kDa \| 8.93 \| \| *Drechslerella brochopaga* KAF3907314 \| 74.4% (AoPak2) \| 91.353 kDa \| 8.53 \| \| *Neurospora crassa* XP_957292 \| 48.8% (AoPak2) \| 91.717 kDa \| 9.56 \| \| *Magnaporthe oryzae* KAI6352866 \| 50.6% (AoPak2) \| 91.182 kDa \| 9.4 \| \| *Arthrobotrys entomopaga* KAF3901726 \| 77.9% (AoPak2) \| 91.271 kDa \| 8.83 \| \| *Arthrobotrys flagrans* RVD90321 \| 94.2% (AoPak2) \| 103.058 kDa \| 9.29 \| \| *Saccharomyces cerevisiae* NP_014101 \| 20.37% (AoPak2) \| 93.909 kDa \| 9.51 \| |
| --- | --- | --- | --- | --- | --- | --- | --- | --- | --- | --- | --- | --- | --- | --- | --- | --- | --- | --- | --- | --- | --- | --- | --- | --- | --- | --- | --- | --- | --- | --- | --- | --- | --- | --- | --- | --- | --- | --- | --- | --- | --- | --- | --- | --- | --- | --- | --- | --- | --- | --- | --- | --- | --- | --- | --- | --- | --- | --- | --- | --- | --- | --- | --- | --- | --- | --- | --- | --- | --- | --- | --- | --- | --- | --- | --- | --- | --- | --- | --- | --- | --- | --- | --- | --- | --- | --- | --- | --- | --- | --- | --- | --- | --- | --- | --- | --- | --- | --- | --- | --- | --- | --- |
| **Table S3. The partial properties of orthologous Rho-GAPs.** The sequence similarity of AoRho-GAPs with other orthologs from different fungi was analyzed using Geneious 4.8.5 software.   \| Fungal strain and gene ID \| Similarity \| Molecular weight \| Isoelectric point \| \| --- \| --- \| --- \| --- \| \| *Arthrobotrys oligospora* AOL_s00110g81 \| 100% (AoRga1) \| 80.951 kDa \| 10.24 \| \| *Arthrobotrys entomopaga* KAF3915872 \| 73.3% (AoRga1) \| 78.451 kDa \| 10.09 \| \| *Arthrobotrys flagrans* RVD89261 \| 92.5% (AoRga1) \| 80.212 kDa \| 10.28 \| \| *Dactylella cylindrospora* KAF3929848 \| 71.9% (AoRga1) \| 76.428 kDa \| 10.05 \| \| *Drechslerella brochopaga* KAF3906977 \| 74.9% (AoRga1) \| 78.437 kDa \| 10.21 \| \| *Saccharomyces cerevisiae* NP_009819 \| 20.27% (AoRga1) \| 74.619 kDa \| 8.6 \| \| *Arthrobotrys oligospora* AOL_s00076g167 \| 100% (AoRga2) \| 96.266 kDa \| 6.18 \| \| *Arthrobotrys flagrans* RVD83461 \| 96.3% (AoRga2) \| 95.788 kDa \| 6.24 \| \| *Drechslerella brochopaga* KAF3915818 \| 85.6% (AoRga2) \| 94.870 kDa \| 6.37 \| \| *Dactylella cylindrospora* KAF3937633 \| 85.2% (AoRga2) \| 94.174 kDa \| 6.56 \| \| *Arthrobotrys entomopaga* KAF3924723 \| 86.8% (AoRga2) \| 83.086 kDa \| 6.56 \| \| *Saccharomyces cerevisiae* NP_428268 \| 45.3% (AoRga2) \| 82.209 kDa \| 5.58 \| \| *Pyricularia oryzae* KAH8847618 \| / \| 99.496 kDa \| 6.41 \| \| *Pyricularia oryzae* XP_003712208 \| / \| 99.482 kDa \| 6.41 \| \| *Pyricularia oryzae* KAH8847619 \| / \| 90.331 kDa \| 6.64 \| \| *Neurospora crassa* XP_958756 \| / \| 97.798 kDa \| 6.49 \| \| *Saccharomyces cerevisiae* NP_012052 \| / \| 90.109 kDa \| 6.31 \| \| *Beauveria bassiana* XP_008600515 \| / \| 93.899 kDa \| 6.40 \| \| *Beauveria bassiana* KAF1730978 \| / \| 93.885 kDa \| 6.40 \| |

**Table S4.** **Primers used in this study.**

| **Gene name** | **Gene ID** | **Primer sequence** | **Function description** |
| --- | --- | --- | --- |
| *Aopak1* | AOL_s00004g340 | F-GTAACGCCAGGGTTTTCCCAGTCACGACGACCTAGCGTCGATTTCAGGC | 5' homologous arm |
|  |  | R-ATCCACTTAACGTTACTGAAATCTCCAACACCTCGTTTCGTGCCAGATT | 5' homologous arm |
| *Aopak1* | AOL_s00004g340 | F-CTCCTTCAATATCATCTTCTGTCTCCGACTCAAGCCGTCGCCAGTATTC | 3' homologous arm |
|  |  | R-GCGGATAACAATTTCACACAGGAAACAGCTGCGTAACCTGAGAAATGCG | 3' homologous arm |
| *Aopak1* | AOL_s00004g340 | F-ATCGGGCGGTACTTCATA | Colony verification |
|  |  | R-TTGGGTGGGTTGTTGTTT | Colony verification |
| *Aopak1* | AOL_s00004g340 | F-CCTCACAACCCTCCCTT | probe |
|  |  | R-TTGACCGCAATCTACCC | probe |
| *Aorga1* | AOL_s00110g81 | F-GTAACGCCAGGGTTTTCCCAGTCACGACGTCTTCAAATCGCGGCCTCTC | 5' homologous arm |
|  |  | R-ATCCACTTAACGTTACTGAAATCTCCAACTAGCGGTCCAATCCCGAAAG | 5' homologous arm |
| *Aorga1* | AOL_s00110g81 | F-CTCCTTCAATATCATCTTCTGTCTCCGACCGGGATTTCAGAGCAACCCT | 3' homologous arm |
|  |  | R-GCGGATAACAATTTCACACAGGAAACAGCCTGTCAGTTTCTCACGCCCT | 3' homologous arm |
| *Aorga1* | AOL_s00110g81 | F-ATCATTCGGTGAATTTAGGC | Colony verification |
|  |  | R-TTGTTTGGCAGTGGTGTT | Colony verification |
| *Aorga1* | AOL_s00110g81 | F-AACACCACTGCCAAACAA | probe |
|  |  | R-TGCCGAAGAGTAATATGAAA | probe |
| pCSN44 vector | pCSN44 | F-GTTGGAGATTTCAGTAACGTTAAGTGGAT | Amplify the *hph* |
|  |  | R-GTCGGAGACAGAAGATGATATTGAAGGAGC | Amplify the *hph* |
| *Aopka1* | AOL_s00004g340 | F-ATGGCCATGGAGGCCGAATTCATGCCGATGGAGGACTCCG | Amplify the gene (pGBKT7) |
|  |  | R-TCGACGGATCCCCGGGAATTCCTAACGCTGTTGATGTGCGC | Amplify the gene (pGBKT7) |
| *Aorga1* | AOL_s00110g81 | F-ATGGCCATGGAGGCCGAATTCATGTCGCTCGAAAAACCGC | Amplify the gene (pGBKT7) |
|  |  | R-TCGACGGATCCCCGGGAATTCTCAATTCACACCCGCATTGC | Amplify the genes (pGBKT7) |
| *Aocdc42* | AOL_s00043g439 | F-GCCATGGAGGCCAGTGAATTCATGGTTGTCGCGACCATAAAATG | Amplify the genes (pGADT7) |
|  |  | R-ATGCCCACCCGGGTGGAATTCTTATAACAAGACGCATTTGGACTTCC |  |
| *Aorac* | AOL_s00079g171 | F-GCCATGGAGGCCAGTGAATTCATGGGACCGAAACCCATTC | Amplify the genes (pGADT7) |
|  |  | R-ATGCCCACCCGGGTGGAATTCTAGAATGGTGCAGACCTTGC |  |
| *Aorho* | AOL_s00215g387 | F-GCCATGGAGGCCAGTGAATTCATGACTTACAACCCAGGTCC | Amplify the genes (pGADT7) |
|  |  | R-ATGCCCACCCGGGTGGAATTCTAAAAGGACACAGCAGCCG |  |
| *Aopka1* | AOL_s00004g340 | F-GCGAATTTACAATTGAGCCAAC | RT-PCR |
|  |  | R-GAGGTTGTTCATCTGATTGCTC | RT-PCR |
| *Aopka2* | AOL_s00079g352 | F-GGAACCATTGAAGGCGTTATAC | RT-PCR |
|  |  | R-CTTAACGTCAACACACAGACAG | RT-PCR |
| *Aorga1* | AOL_s00110g81 | F-GCGTTGTATCCCAGAAATTCAG | RT-PCR |
|  |  | R-ATACTCGCTTATCGTGCAAGTA | RT-PCR |
| *Aocdc42* | AOL_s00043g439 | F-TTTCTCGTCTGTTTCTCGGTTA | RT-PCR |
|  |  | R-CATCTTTTGGCGATTCAGCTTA | RT-PCR |
| *Aorac* | AOL_s00079g171 | F-AAATTTGAGTGTACAGTGCGTC | RT-PCR |
|  |  | R-GGCATTGGTGGTGTAACTAATC | RT-PCR |
| *Aorho* | AOL_s00215g387 | F-CTCAAGAAAGATCTTCGCGATG | RT-PCR |
|  |  | R-CTGCATTCCATGTATTTCCGAG | RT-PCR |
| - | AOL_s00075g8 | F-TTGCTACTTTACTGCCCCTTG | RT-PCR |
|  |  | R-TCTTCAGCTTGAGTCCGGTT | RT-PCR |
| - | AOL_s00112g42 | F-CTGGCTCTTGGCCTACTTTG | RT-PCR |
|  |  | R-AGGAGGTTGACGGTCTCCTT | RT-PCR |
| - | AOL_s00054g992 | F-TCCGCAACTTCAAGAGTGTG | RT-PCR |
|  |  | R-CGTTGGCTTCCTCGTTAGAG | RT-PCR |
| - | AOL_s00076g4 | F-CCATGGTGTTGGAAGGAAAT | RT-PCR |
|  |  | R-GTCTGTTCAGCGTACGTAGT | RT-PCR |
| - | AOL_s00215g702 | F-GTCGCCGCTGACTTAACTGT | RT-PCR |
|  |  | R-ATAATTGCTGATTCGCTGGG | RT-PCR |
| *Aoglr* | AOL_s00054g13 | F-CGAAAAGTCTGAATCGGGTGA | RT-PCR |
|  |  | R-GCTCCACTTTGCCACATACATC | RT-PCR |
| *Aogld* | AOL_s00043g396 | F-TCTGCCATACTGATCAGTACAC | RT-PCR |
|  |  | R-ATTTGTCTTTCCACTCTTGCAG | RT-PCR |
| *Aothi* | AOL_s00076g248 | F-CTACTCCTCTGAAGCTGCTC | RT-PCR |
|  |  | R-TCCATAGCTGCCATCATCGT | RT-PCR |
| *Aoper* | AOL_s00215g326 | F-CACCATCCGCTCTGTCTTCA | RT-PCR |
|  |  | R-GACGGCATCCTCGGTCTTGA | RT-PCR |
| *Aocat* | AOL_s00173g374 | F-TCCCCATCCTCATCCATACG | RT-PCR |
|  |  | R-GATAGCGGGCATTTCTTTCC | RT-PCR |
| *Aochs-1* | AOL_s00078g76 | F-GCCACTCTGCCATCTTTAGC | RT-PCR |
|  |  | R-GCATCTTCACCCGCACCAGT | RT-PCR |
| *Aotrs* | AOL_s00097g268 | F-CACGTCCATATCACCCTCGA | RT-PCR |
|  |  | R-GTGGTATCGGCGACAGTTTC | RT-PCR |
| *Aoglu* | AOL_s00083g375 | F-GTCATCCTCAAGAACGTCGC | RT-PCR |
|  |  | R-ATATGGAAAGTTGGCCGTGC | RT-PCR |
| *Aogls* | AOL_s00054g491 | F-AGCTCTGTTCTGGTGATGCT | RT-PCR |
|  |  | R-GATGTTTCGCCAAGGACTCC | RT-PCR |
| *Aohex* | AOL_s00112g89 | F-ACCCTTCTTGACCTCGCTG | RT-PCR |
|  |  | R-GAGACGGCGGATGAGTTTTC | RT-PCR |
| *AobrlA* | AOL_s00097g514 | F-TTGAGGCCTCGATCCGTAGA | RT-PCR |
|  |  | R-AGGTAGATGGCGCTGTTACG | RT-PCR |
| *AoflbC* | AOL_s00007g157 | F-CTCTCCGGCAAAGACAATCG | RT-PCR |
|  |  | R-GTCGACTGAGGATAGTAGCT | RT-PCR |
| *AofluG* | AOL_s00043g361 | F-GATTCCAGTCCCGTGAATTC | RT-PCR |
|  |  | R-GCTAAGGAGAGGATGGGCAT | RT-PCR |
| *AomedA* | AOL_s00210g120 | F-TCCGGCCCAATGATTCAGAA | RT-PCR |
|  |  | R-AGATCGCAGGAACATGGTGA | RT-PCR |
| *AoveA* | AOL_s00169g18 | F-AAGCTACACCCAATCAACGC | RT-PCR |
|  |  | R-TTGCGATGCTGACGATCTTG | RT-PCR |
| *AovelB* | AOL_s00054g811 | F-ATTCCGCAACTTCTCCCTCA | RT-PCR |
|  |  | R-GGCATGTTTGGATTCTGGGG | RT-PCR |
| *AowetA* | AOL_s00173g221 | F-TTACATGCCACCCCAAGTCC | RT-PCR |
|  |  | R-CAATTGCAACTGCGTCCACA | RT-PCR |
| *AovosA* | AOL_s00054g700 | F-CAAACCACCCACCACCAAAT | RT-PCR |
|  |  | R-GGATGGACAGGAGAAGGACC | RT-PCR |
| *AonsdD* | AOL_s00075g211 | F-ATTACGGCCGCCTAGTAGTC | RT-PCR |
|  |  | R-CTCGTTTGGACCTGGTTGTG | RT-PCR |
| *Aohyp* | AOL_s00006g570 | F-GCGGATCCAACATGAAGCTT | RT-PCR |
|  |  | R-GGTTGACAACTGGGATGCTG | RT-PCR |
